# Distribution- and anchor-based methods to determine the minimally important difference on patient-reported outcome questionnaires in oncology: a structured review

**DOI:** 10.1186/s12955-018-1055-z

**Published:** 2018-12-11

**Authors:** Ahmad Ousmen, Célia Touraine, Nina Deliu, Francesco Cottone, Franck Bonnetain, Fabio Efficace, Anne Brédart, Caroline Mollevi, Amélie Anota

**Affiliations:** 1 Methodology and Quality of Life in Oncology Unit (INSERM UMR 1098), University Hospital of Besançon, Besançon, France; 2 Montpellier Cancer Institute (ICM) – Val d’Aurelle, University of Montpellier, Montpellier, France; 3 Italian Group for Adult Hematologic Diseases (GIMEMA), Data Center and Health Outcomes Research Unit, Rome, Italy; 4 French National Platform Quality of Life and Cancer, Besançon, France; 5 Institut Curie, Supportive Care Department, Psycho-Oncology Unit, Paris, France; 6 University Paris Descartes, Psychopathology and health process laboratory EA 4057, Boulogne-Billancourt, France; 7 IRCM, University of Montpellier, ICM, INSERM, Montpellier, France

**Keywords:** Patient-reported outcomes, Minimally important difference, Anchor-based approach, Distribution-based approach

## Abstract

**Background:**

Interpretation of differences or changes in patient-reported outcome scores should not only consider statistical significance, but also clinical relevance. Accordingly, accurate determination of the minimally important difference (MID) is crucial to assess the effectiveness of health care interventions, as well as for sample size calculation. Several methods have been proposed to determine the MID. Our aim was to review the statistical methods used to determine MID in patient-reported outcome (PRO) questionnaires in cancer patients, focusing on the distribution- and anchor-based approaches and to present the variability of criteria used as well as possible limitations.

**Methods:**

We performed a systematic search using PubMed. We searched for all cancer studies related to MID determination on a PRO questionnaire. Two reviewers independently screened titles and abstracts to identify relevant articles. Data were extracted from eligible articles using a predefined data collection form. Discrepancies were resolved by discussion and the involvement of a third reviewer.

**Results:**

Sixty-three articles were identified, of which 46 were retained for final analysis. Both distribution- and anchor-based approaches were used to assess the MID in 37 studies (80.4%). Different time points were used to apply the distribution-based method and the most frequently reported distribution was the 0.5 standard deviation at baseline. A change in a PRO external scale (*N* = 13, 30.2%) and performance status (*N* = 15, 34.9%) were the most frequently used anchors. The stability of the MID over time was rarely investigated and only 28.2% of studies used at least 3 assessment timepoints. The robustness of anchor-based MID was questionable in 37.2% of the studies where the minimal number of patients by anchor category was less than 20.

**Conclusion:**

Efforts are needed to improve the quality of the methodology used for MID determination in PRO questionnaires used in oncology. In particular, increased attention to the sample size should be paid to guarantee reliable results. This could increase the use of these specific thresholds in future studies.

**Electronic supplementary material:**

The online version of this article (10.1186/s12955-018-1055-z) contains supplementary material, which is available to authorized users.

## Introduction

The use of patient-reported outcomes (PRO), including health-related quality of life (HRQOL), in cancer clinical trials has substantially increased over the years [1]. PROs are critical to fully understand overall treatment effectiveness and to establish the benefit of a given experimental drug over the standard of care in a particular cancer population [2, 3]. Thus, assessment and analysis of PRO data must be carried out in compliance with a rigorous and appropriate methodology to ensure robust interpretation of the results [4].

The interpretation of PRO scores and their clinical importance is a major challenge, in terms of both clinically relevant score differences between two measurement times and two treatment arms [5]. A statistically significant result may not be clinically relevant, as it should also reflect changes or differences that are meaningful for the patient, i.e., they should take into account a minimally important difference (MID). The MID was defined by Jaeschke et al. as “the smallest change in an outcome that a patient would identify as important” [6]. Hence, the determination of the MID is crucial in order to assess the effectiveness of health care interventions, as well as for sample size calculation when HRQOL is the primary or co-primary endpoint in clinical trials.

Different methods have been proposed to determine the MID. These methods are generally grouped into two categories, namely anchor-based and distribution-based approaches [7, 8]. The anchor-based approaches use an external indicator, called an “anchor”, and differences can be determined either cross-sectionally (differences between clinically-defined groups at one time point) or longitudinally (change in the scores of a single group over time). The anchor can be either an objective measure (e.g., Karnofsky or ECOG performance status) or a subjective measure, generally reflecting the patient’s point of view, which is of interest (for example, the patient rating of change). Distribution-based approaches are based on statistical criteria from the PRO scores. These approaches include fractions of the standard deviation (SD) of PRO scores, the effect size [9], and the standard error of measurement (SEM) [10] as estimates for the MID. Distribution-based approaches have the advantage of simplicity of use, since they do not require an external criterion. However, they produce similar MID results for both deterioration and improvement. This simplifies the interpretation but may be questionable, since a larger MID is often observed for deterioration than for improvement [11].

Some recommendations have been proposed regarding the best method to apply, depending on the design of the study. For instance, analysis must rely primarily on relevant patient-based and clinical anchors [12]. Moreover, both distribution- and anchor-based approaches remain the most commonly used methods to determine the MID [13]. However, robust and reliable determination of the MID remain challenging. In fact, due to the longitudinal design often used in MID analyses, a potential response shift effect may bias the results. The impact of the response shift effect on the longitudinal analysis of PRO is well established and has been widely studied [14]. However, studies investigating the impact of response shift effect on MID determination remain sparse [15]. Another important possible limitation of studies aiming to determine the MID is the sample size. Indeed, most studies aiming to explore the MID on a given PRO questionnaire use data from an existing cohort or randomized clinical trial. Thus, the volume of available data may not be sufficient to provide a reliable MID, in particular due to the number of possible categories for the anchor.

To date, longitudinal studies in oncology generally used the thresholds proposed by Osoba et al. in 1998 [5] for HRQOL for the interpretation of results, i.e. an MID of 5 or 10 points. However, these thresholds were obtained only on data collected with the European Organisation for Research and Treatment of Cancer (EORTC) QLQ-C30 cancer-specific questionnaire. A more recent meta-analysis proposed specific thresholds for each HRQOL scale of the EORTC QLQ-C30, and for each direction, i.e. improvement or deterioration [16]. Other studies proposed MID for specific cancer site questionnaires, such as the EORTC QLQ-BN20 for brain cancer [17], but few studies use these specific thresholds to interpret HRQOL results.

In this context, the objective of this structured review was to assess the most common practices used by the distribution and anchor-based approaches to determine the MID for PRO questionnaires in oncology, as well as the characteristics and the possible limitations relative to each approach.

## Methods

### Search and selection strategy

A systematic literature search was performed in the Pubmed database, of all articles published between January 2000 and May 2018. Eligible studies included original articles aiming to determine the MID of self-administered questionnaires in cancer, using distribution- and/or anchor based approaches. Only static questionnaires were considered, i.e. questionnaires that have a fixed number of questions answered by patient. It means, all patients will answer to the same questions in the same order. Accordingly, computer adaptive tests were not included in this review. Indeed, all studies using Item Response Theory models were not included since these types of models are very specific and results could not be directly comparable to studies using the summary score. All non-cancer studies were excluded as well as reviews and meta-analysis. The following search strategy was used:

(MCID OR MID OR MCIDs OR MIDs OR “minimal clinically important” OR “minimum clinically important” OR “minimally clinically important” OR “minimal important” OR “minimally important” OR “clinically meaningful” OR “meaningful change” OR “meaningful changes” OR “meaningful difference” OR “meaningful differences” OR “cutoff score” OR “cutoff scores”) AND (“quality of life” OR QoL OR “patient-reported outcomes” OR “patient-reported outcome” OR PRO OR PROs OR HRQOL OR symptom OR symptoms) AND (“anchor-based” OR “distribution-based” OR anchored OR anchor) AND cancer AND (“2000/01/01”[Date - MeSH]: “2018/05/31”[Date - MeSH])

### Data extraction

Two reviewers (A.O., C.T.) independently screened first titles and abstracts and secondly full paper to identify relevant articles. Then, they independently extracted information from eligible studies using a predefined data extraction form (DEF). All discrepancies were resolved by mutual consensus. In case of disagreement, a third author (A.A.) was consulted to reach a final consensus.

This literature review was performed according to the Preferred Reporting Items for Systematic Reviews and Meta-Analysis (PRISMA) statement guidelines [18] and the following details were extracted:

- General items, namely, year of publication, number of patients, disease stage, type of study (randomized clinical trial, prospective cohort, or other), study location, international and multicenter study or not.
- Items regarding the PRO assessment, including the name of the PRO questionnaire for which the MID was determined, the time windows and number of measurement times considered for the MID determination.
- Items regarding the MID determination, including the term used for the MID designation (e.g., minimal important difference or minimal clinically important difference), name and number of PRO scales analyzed, level for statistical significance if appropriate, type (e.g. distribution or anchor based approach) and number of approaches used (1 or 2), and the design considered (cross-sectional or longitudinal). Regarding the anchor-based approach, information on the number and type of anchors used, the threshold considered to qualify the minimal important change, whether the correlation between the anchor and HRQOL/PRO scores was assessed, and the minimum number of patients included in each category of the anchor were collected. For the distribution-based approach, different criteria were extracted. Finally, whatever the method(s) used, we also recorded the recommendations proposed for the MID to be used in future studies, the limitations highlighted by the authors, and the potential risks of bias (for instance, missing data, bias in the selection population and bias in the statistical analysis (e.g. correlation between anchor and HRQOL score not assessed for longitudinal studies)).

### Data analysis

A descriptive analysis of eligible publications was performed. Qualitative variables were summarized by tabulating frequency distribution and percentages and quantitative data by median and interquartile range (IQR). Analyses were performed using SAS version 9.3. (SAS Institute Inc., Cary, NC, USA).

## Results

**Fig. 1:**
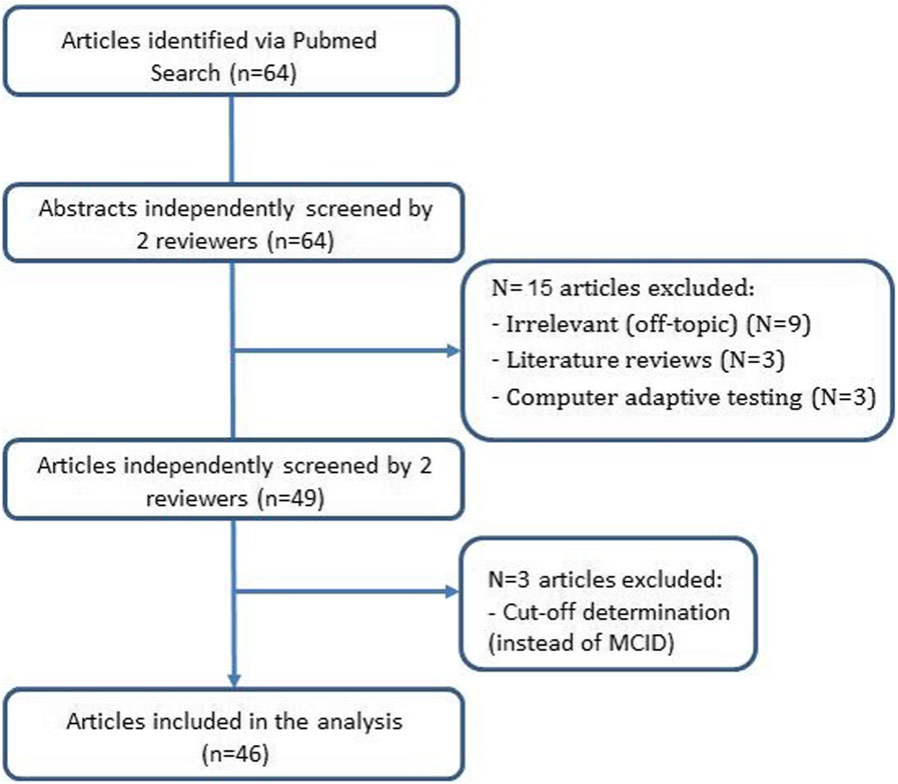
Flow chart of the study selection procedure

The initial search identified a total of 64 studies (Fig. 1). After screening of the title and abstract by the 2 reviewers, 15 studies were excluded because they were not relevant to the subject (*N* = 9), were not original papers (*N* = 3) or reported computer adaptive testing (*N* = 3). After reading the full text of the remaining 49 articles, three additional articles were excluded as they explored cut-off scores and not MID. Thus, a total of 46 studies were finally included in this review [13, 17, 19–62].

### Characteristics of the studies included

The sample sizes of the studies included ranged from 50 to 3770. The general results are presented in Table 1. Among the 46 articles retained for analysis, 20 (43.5%) enrolled patients with metastatic or advanced cancer, 5 (10.9%) included patients with localized cancer, and 12 (26.1%) included both. The majority of studies were prospective cohort studies (*N* = 23, 50%) and 10 were randomized clinical trials (21.7%). Eighteen studies (39%) assessed the MID of an EORTC questionnaire, specifically, the EORTC QLQ-C30 questionnaire (*N* = 10, 21.7%), the QLQ-C15-PAL (*N* = 2, 4.3%) and EORTC specific modules, such as the EORTC QLQ-BN20 brain cancer module (*N* = 6, 13%). Thirteen studies (26.1%) evaluated the MID of a Functional Assessment of Cancer Therapy (FACT) questionnaire, of which 8 (61.5%) used a FACT questionnaire specific to the cancer site, including the FACT-M for melanoma patients (*N* = 2, 4.3%).

**Table 1:** General information for all studies selected (*N* = 46)

	Number	Percent
Disease stage		
Metastatic / Advanced	20	43.5
Non metastatic / Local	5	10.9
Both	12	26.1
Unclear or not reported	9	19.5
Type of study		
Randomized clinical trials	10	21.7
Prospective cohort study	23	50
Other	13	28.3
International study		
Yes	9	19.6
No	37	80.4
Multicenter study		
Yes	22	47.8
No	13	28.3
Unclear or not reported	11	23.9
Questionnaires		
EORTC QLQ-C30	10	21.7
EORTC QLQ-C15-PAL	2	4.3
EORTC specific modules	6	13.0
FACT-G	1	2.2
Other FACT questionnaires	13	21.7
Other multidimensional questionnaires	8	17.4
Other specific PRO questionnaires	13	28.2

Other questionnaires assessed a specific PRO domain such as pain or fatigue. For example, 4 studies explored the MID of fatigue PRO questionnaires, such as the FACT-F (*N* = 1, 2.3%), the Multidimensional Fatigue Inventory-20 (MFI-20) (*N* = 1, 2.3%), the Multidimensional Fatigue Symptom Inventory-Short Form (MFSI-SF) (*N* = 1, 2.3%) and 1 study (2.3%) used three different instruments (the cancer fatigue scale (CFS), the Schwartz Cancer Fatigue Scale-revised (SCFS-r), and the fatigue symptom inventory (FSI)). Two other studies (4.4%) addressed the MID of the Brief Pain Inventory or its short form. Finally, 5 studies (10.9%) assessed the MID of generic questionnaires for cancer patients, for example the EuroQoL EQ-5D (*N* = 3, 6.5%).

### Statistical analysis of the MID

General results regarding the MID determination are presented in Table 2. Several terminologies were used to identify the MID. The MID was the most used acronym t with 29 studies (63%), and referring to “Minimally important difference” (*N* = 16, 34.8%) or “Minimal important difference” (*N* = 13, 28.2%). The second used term was the MCID referring to “Minimal clinically important difference” in 16 studies (34.8%), to “Minimum clinically important difference” in 2 studies (4.3%) and to “Minimal clinical important difference” in one study (2.2%). The last used term was the MIC referring to “Minimal important change” in only one study (2.2%).

**Table 2:** General results regarding the minimally important difference (MID) determination (*N* = 46)

	Number	Percent
Approach used for MID determination		
Distribution-based only	3	6.5
Anchor-based only	6	13.1
Both distribution- and anchor-based	37	80.4
Term used to design the MID		
MID, referring to:	29	63
Minimally important difference	16	34.8
Minimal important difference	13	28.2
MCID, referring to:	19	41.3
Minimal clinically important difference	16	34.8
Minimum clinically important difference	2	4.3
Minimal clinical important difference	1	2.2
MIC, referring to:	1	2.2
Minimal important change	1	2.2
Number of assessment timepoints		
1	2	4.4
2	31	67.4
3	6	13
≥ 4	7	15.2
Time interval between the assessment times		
2 days	1	2.2
1 month	7	15.2
3–6 months	6	13
1 year	2	4.4
Based on clinical relevance	3	6.5
Multiple with a maximum interval of 6 months	14	30.4
Multiple with a maximum interval > 1 year	5	10.9
Multiple intervals based on clinical relevance	2	4.4
Floor and ceiling effects studied		
Yes	7	15.2
Floor and ceiling effects detected (< 15%)	2	28.6
Floor and ceiling effects no detected (< 15%)	4	57.1
Not reported	1	14.3
No	39	84.8
Recommendations for futures studies		
Yes	16	34.8
No	23	50
Unclear	7	15.2
Limitations highlighted by authors		
Yes	42	91.3
No	4	8.7
Possible risk of bias		
Missing data on the HRQOL/PRO measures	16	40
Bias in the selection of the population	17	42.5
Bias in the statistical analysis	5	10.9

Three studies (6.5%) used only the distribution-based approach, 6 studies (13.1%) used only the anchor-based approach, and 37 studies (80.4%) used both. Concerning the number of assessment times, 2 studies (4.4%) used only one measurement time for the determination of the MID, 31 studies (67.4%) used two measurement times and 13 studies (28.2%) used at least three measurement times. Only one study explored the impact of the occurrence of the response shift effect on the MID determination over time. The time interval between two assessment times varied from 2 days (*N* = 1, 2.5%) to more than 1 year (*N* = 5, 12.5%). For most of the studies, the time interval between two assessment was between 1 and 6 months (*N* = 27, 58.7%). Floor and ceiling effects are studied in 7 studies (15.2%). For 4 studies (57.1%), the range of floor and ceiling effects is < 15%. For only 2 studies (28.6%), the range was ≥15% and not reported in one study (14.3%).

#### Distribution-based approach

Results of distribution- and anchor-based approaches are presented in Table 3. A total of 40 studies (87%) used distribution-based approaches. The reported criteria (fraction of SD or SEM) were extracted: at baseline, after follow-up and between two measurement times. The most commonly used distribution was the 0.5 SD at baseline (*N* = 36, 90%) followed by the SEM at baseline (*N* = 31, 77.5%). Twenty-five studies (62.5%) reported the 0.3 SD at baseline and 12 studies (30%) used the 0.2 SD at baseline. Among the other reported criteria, the 0.3 or 0.5 SD at follow-up was reported by 14 studies (35%) and the 0.3 or 0.5 SD of a change by respectively 8 (20%) and 7 studies (17.5%).

**Table 3:** Results of distribution and anchor based approaches (*N* = 46)

	Number	Percent
Distribution-based approach (*N* = 40)		
Distribution-reported		
0.2 SD at baseline	12	30
0.3 SD at baseline	25	62.5
0.5 SD at baseline	36	90
SEM at baseline	31	77.5
0.2 SD at follow-up	7	17.5
0.3 SD at follow-up	14	35
0.5 SD at follow-up	14	35
SEM at follow-up	13	32.5
0.2 SD of change	2	5
0.3 SD of change	8	20
0.5 SD of change	7	17.5
SEM of change	5	12.5
Anchor-based approach (*N* = 43)		
Study design		
Cross-sectional	3	7.1
Longitudinal design	39	92.9
Number of anchors		
1	23	53.5
2	8	18.6
3	3	7
4	3	7
≥ 5	6	13.9
Anchors		
Overall rating of change	9	20.93
Anchor derived from an external questionnaire	13	30.2
Anchor derived from one dimension of the questionnaire studied	8	18.6
Performance status	15	34.9
MMSE	1	2.3
Weight loss	1	2.3
Other	10	23.3
Correlation checked between anchor and the studied questionnaire		
Yes	32	76.2
No	9	21.4
Criteria used to detect a moderate correlation (*N* = 32)		
≥ 0.3	15	46.9
≥ 0.4	1	3.1
≥ 0.5	1	3.1
Not reported	15	46.9
Minimum N by anchor category		
≤ 20	16	37.2
> 20	18	41.9
Not reported	9	20.9

*SD* standard deviation, *SEM* standard error of measurement, *MMSE* mini mental state examination

#### Anchor-based approach

Forty-three studies (93.5%) used the anchor-based approach to estimate the MID. Among them, 39 studies (92.9%) used a longitudinal design regarding the anchor while only 3 studies (7.1%) used a cross-sectional design. The correlation between the anchor and the PRO scores was assessed in 32 studies (74.4%). The most used threshold to detect a moderate correlation between anchor and HRQOL scores was 0.3 in 15 studies (46.9%). Every dimension with a correlation greater than this threshold (|r| ≥ 0.3) was retained for the MID determination. For 15 studies (46.9%), the correlation has been calculated but there was no specified threshold for the retained scales that have been used in the MID determination. The minimal number of patients analyzed by category of anchor was less than 20 in 16 studies (37.2%). For instance, 13 and 7 patients were used for one category of the anchor to qualify deterioration and improvement respectively in two studies.

Twenty-three studies (53.5%) used only one anchor, while the other studies used from 2 (18.6%) to more than 5 anchors (13.9%). The median number of anchors used was 1 (IQR 1–3). Various anchors were used to assess the MID. Some were subjective, i.e. patient centered and reflecting the patient’s perception of change or HRQOL, while others were more objective, reflecting clinical or biological measures or the physician’s assessment of change. A total of 28 studies (65.1%) used at least one patient-centered anchor and 15 studies (34.9%) exclusively used some objective anchors (Table 4).

**Table 4:** Information including number of patients included and analyzed and anchor used for each study selected (*N* = 46)

Reference	Number of patients included	Number of patients included in the analysis (range for multiple analyses)	Questionnaires on which the MID was determined	Anchor
Askew, R.L. (2009) [19]	273	163	FACT-M	Performance status
Bédard, G. (2014) [21]	369	367 to 369	EORTC QLQ-C30	Global HRQOL dimension of the QLQ-C30
Bédard, G. (2016) [22]	276	276	EORTC QLQ-C15-PAL	Global HRQOL dimension of the QLQ-C15 PAL
Bédard, G. (2016) [22]	421	197 to 276	EDMONTON SYMPTOM ASSESSMENT SYSTEM (ESAS)	Well-being dimension of the ESAS
Bharmal, M. (2017) [23]	88	70	FACT-M	Percentage change in tumor size
Binenbaum, T. (2014) [24]	1011	329 to 631	UW-QOLQ, EORTC QLQ-C30, QLQ-H&N35	No anchor used
Cella, D. (2009) [26]	809	809	FACT-P	Performance status
Cella, D. (2002) [13]	50;	50 to 2402	FACT-AN	Performance status; hemoglobin level
Cella, D. (2002) [25]	599	573	FACT-L	Best overall response to treatment, time to disease progression
Chan, A. (2018) [27]	257	201	MFSI-SF	Fatigue dimension of the QLQ-C30
Cheung, Y.T. (2014) [28]	330	220	FACT-Cog	Cognitive dimension of the QLQ-C30
Den Oudsten, B.L. (2013) [29]	606	355	WHOQOL-100	QoL of the WHOQoL
Eton, D.T. (2007) [32]	92	91	FACT-G, FACT-Lung Symptom Index-12	Performance status
Eton, D.T. (2006) [30]	209	209	FACT-BRM	Clinical distinct groups using performance status
Eton, D.T. (2004) [31]	771	128 to 643	FACT-B	Performance status, physician assessment of current pain, and response to treatment
Granger, C.L. (2015) [34]	69	69	Physical Activity Scale For The Elderly (PASE)	No anchor used
Granger, C.L. (2015) [33]	56	63 to 66	6-Minute Walk Distance	Physical functioning dimension of the QLQ-C30
Hong, F. (2013) [35]	765	627	EORTC QLQ-C30	Patient’s rating of change
Hui, D. (2016) [36]	796	792 to 795	Edmonton Symptom Assessment System (ESAS)	Patient’s rating of change
Jayadevappa, R. (2012) [37]	602	528	SF-36, UCLA Prostate Cancer Index (PCI)	A patient-reported physical signs/symptoms (more tired or worn out than usual)
Kemmler, G. (2010) [38]	187	160	EORTC QLQ-C30	Patient’s rating of change
Lemieux, J. (2007) [39]	235	133	EORTC QLQ-C30, POMS, MAC, IES, PAIS, PAIN	No anchor used
Liu, H. (2015) [40]	2440	≤ 246	2 single-item questions	Patient’s rating of change
Maringwa, J.T (2011) [41]	941	420 to 572	EORTC QLQ-C30, QLQ-BN20	Performance status; MMSE
Maringwa, J.T (2011) [41]	812	410 to 519	EORTC QLQ-C30	Performance status; weight change
Mathias, S.D. (2011) [42]	2049	1564	Brief Pain Inventory-Short Form (BPI-SF)	BPI-SF current pain and EQ-5D index score
Mouysset, J.L. (2016) [43]	1262	510	FACT-F	VAS of fatigue
Ousmen, A. (2016) [62]	381	74 to 260	EORTC QLQ-C30; QLQ-BR23	Patient’s rating of change
Pickard, A.S. (2007) [44]	534	534	EQ-5D	Performance status
Purcell, A. (2010) [45]	210	157 to 199	MFI-20	VAS of the EQ-5D; performance status; treatment impact on fatigue
Raman, S. (2016) [46]	298	201 to 204	EORTC QLQ-BM22, QLQ-C15-PAL	Global HRQOL dimension of the QLQ-C15 PAL
Raman, S. (2018) [47]	850	360 to 375	EORTC QLQ-C30, BPI	Global HRQOL dimension of the QLQ-C30
Sagberg, L.M. (2014) [48]	173	142 to 164	EQ-5D 3 L	Performance status
Shun, S.C (2007) [49]	243	148	3 fatigue instruments	Patient’s rating of change
Skolarus, T.A. (2015) [50]	1201	1201	EPIC-26	Item from the service satisfaction scale for cancer care scale
Steel, J. L. (2006) [51]	158	158	FACT-HEP	Alpha-fetoprotein, alkaline phosphate and hemoglobin levels, survival
Tamminga, S.J. (2014) [52]	53	43	Work Limitations Questionnaire (WLQ)	Patient’s rating of change
Tsiplova, K. (2016) [53]	3770	3765	EQ-5D	Global health question
Tuomi, L. (2016) [54]	126	119	Swedish self-evaluation of communication experiences after laryngeal cancer (S-SECEL)	Acceptability of speech in a social context
Wong, E. (2015) [55]	99	77 to 99	EORTC QLQ-BN20	Item 30 of the QLQ-C30 or 15 of QLQ-C15 PAL
Wong, K. (2013) [56]	414	153 to 233	Brief Pain Inventory (BPI)	Pain score of the BPI
Wright, P. (2008) [57]	276	187	Social Difficulty Inventory	Social functioning dimension of the QLQ-C30
Yost, K.J. (2005) [58]	200	144 to 164	FACT-BRM	Patient’s rating of change; performance status
Yost, K.J. (2005) [58]	60	60 to 568	FACT-C	Performance status
Yost, K.J. (2011) [59]	101	88 to 101	PROMIS-cancer scales	Patient’s rating of change; performance status; 21 other anchors
Zeng, L. (2012) [61]	400	88 to 93	EORTC QLQ-C30, QLQ-BM22	Performance status

Regarding patient-centered anchors, 9 studies (20.9%) used the patient’s overall rating of change in HRQOL, or a specific domain, while 18 studies (41.9%) used an anchor derived from a PRO questionnaire. This could either be a PRO scale from the same questionnaire on which the MID was determined (*N* = 8, 18.6%), or an external questionnaire (*N* = 13, 30.2%). For example, the global HRQOL dimension or overall QoL item of the EORTC QLQ-C30 or QLQ-C15-PAL questionnaire was used as an anchor in 5 studies (11.6%), while the MID was determined on other dimensions of the EORTC QLQ-C30 or QLQ-C15PAL. An external item or scale derived from another questionnaire was also used as an anchor in 13 studies (30.2%). For example, a visual analogue scale of fatigue was used in one study as an anchor to determine the MID on the FACT-Fatigue questionnaire. The fatigue dimension of the EORTC QLQ-C30 was also used as an anchor in one study to determine the MID on the MFSI-SF questionnaire.

Regarding clinical anchors, the performance status (either Karnofsky or ECOG) was used in 15 studies (34.9%). Weight loss and the Mini-Mental State Examination (MMSE) score were both used in one study (2.3%).

Studies using the same anchor did not necessarily use the same threshold to qualify the minimal change for the anchor. For example, among the 5 studies using the global HRQOL dimension or its items individually as an anchor, 2 studies used a 10-point difference in the global score as a minimal change and 3 studies used only one item of the overall QoL scale by considering a change of two units (*N* = 2) or one unit (*N* = 1) as the minimal change. When these single items are standardized on a 0 to 100 scale, a change of one unit corresponds to a change of 16.7 points and a change of two units corresponds to a change of 33.3 points. Regarding studies using physician-reported performance status as an anchor, they generally used a 10 point difference for the Karnofsky index and change of one category for the ECOG as a clinically relevant change.

To complement these results, we summarized the information collected for the main questionnaires used in our review, namely the EORTC QLQ-C30 and the FACT questionnaire in the Additional file 1: Table S1. Among the 23 studies using either the EORTC QLQ-C30 or the FACT questionnaire, 18 (78.3%) used both distribution and anchor-based method to determine the MID. Among the 21 studies that used the anchor-based approach to determine the MID for either EORTC QLQ-C30 or FACT questionnaires, 4 studies (19%) determined the MID without distinction between improvement and deterioration.

Sixteen studies (34.8%) proposed recommendations for MID for use in futures studies. In the majority of studies (*N* = 42, 91.3%), some limitations were reported by the authors. Regarding the possible risk of bias, 16 studies (43.2%) were impacted by the occurrence of missing data on the PRO measures; in 17 studies (47.2%), the selection of the population could be subject to a risk of bias, and for 5 studies (19.2%) there was a risk of bias due to the statistical analysis.

## Discussion

The objective of this structured review was to assess the most common practices used by the distribution and anchor based approaches to determine the MID for PRO questionnaires in oncology, and to present the variability of criteria used as well as possible limitations relative to each approach. We limited our research to year 2000 because we think a review of papers published since almost two decades would be reasonable and enough to conduct this review. Eligible studies included original articles aiming to determine the MID of self-administered questionnaires in cancer, using distribution- and/or anchor based approaches.

Using both the distribution and anchor-based approaches, as was the case in the majority of studies (80%), makes it possible to compare results for consistency, to highlight the strengths and weaknesses of each method, and to retain the most appropriate MID value or range to apply in further studies [12].

For the distribution-based approach, several criteria were reported at different assessment times. As already highlighted in previous reviews [63], the most frequently reported criterion was 0.5 SD at baseline, reported in 90% of studies using the distribution-based method. Despite the simplicity and the widespread use of this approach in the determination of the MID, no distinction can be made between improvement and deterioration.

Regarding the anchor-based approach, most studies used a longitudinal design (92.9%). Various anchors were applied, and were either patient- or physician-reported measures, as well as clinical or biological measures with clinical relevance. The most commonly used anchor was a PRO score or item, derived from the questionnaire of interest or from another questionnaire (41.9%). When this anchor was derived from the questionnaire for which the MID was being determined, then the MID on the corresponding dimension could not be assessed. This is the case for example when the overall HRQOL score is used as an anchor to estimate the MID on the QLQ-C30. Moreover, this requires to fix a threshold to qualify the clinically meaningful change on one dimension of the studied questionnaire. Finally, the choice of this kind of anchor could be questionable since the property of external criteria for the anchor is not entirely respected.

Another frequently used patient-centered anchor (used in 20.9% of the studies using the anchor-based approach) is the patient’s overall rating of change. This anchor reflects the patient’s perception of change, but it needs to be planned in the design of the study.

A large proportion of studies also used physician-reported measures such as performance status or MMSE score. These anchors could be considered as objective evaluations of the patient’s health status. However, they may not be appropriate for the assessment of the MID on all HRQOL dimensions. Given that performance status reflects the physical condition of the patient, it is generally correlated to the physical dimensions of HRQOL. Similarly, the MMSE is mostly correlated with the cognitive dimension of HRQOL. Thus, these anchors preclude assessment of the MID for more physiological or emotional dimensions of HRQOL [17]. This also means that several anchors are needed to accurately assess the MID and to check the robustness and complementarity of the results obtained using different anchors [12]. In our review, half the studies (53.5%) using an anchor-based approach used only one anchor. For the majority of the studies, the correlation between the anchor and scores of the studied questionnaire has been checked. The threshold of 0.3 was the most used criteria to detect a moderate correlation. On the other side, an important frequency of studies (46.9%) from those who checked correlation did not reported the criteria to identify a moderate correlation. Generally, checking correlation is important to know to what extent the anchor used is linked to HRQOL measure. Hence, the correlation between the anchor and the PRO scores must be assessed and only dimensions that are significantly correlated with the anchor (correlation coefficient |r| > 0.3) should be analyzed. In this review, 76.2% of the studies using an anchor-based approach verified this correlation.

The majority of the studies with a longitudinal design (58.7%) used a time interval between 1 and 6 months between two consecutive assessments. However, wide variation was observed between studies. A standard period remains to be determined, and further research is warranted to determine a suitable time window. This point is particularly important when the patient’s overall rating of change is used as an anchor, since long periods between assessments could induce a recall bias.

The majority of studies (71.7%) used one or two measurement times to determine the MID, but the stability over time was rarely investigated. For example, a change in PRO score of 5 points could be insignificant for the patients at the time of diagnosis, whereas it might be highly relevant after surgery. Therefore, it is strongly recommended to assess the MID with more than two measurement times [62]. This change in the patients’ perception of HRQOL change over time could reflect the occurrence of a response shift effect [64]. In our review, only one study investigated the impact of the occurrence of response shift on the MID determination using the patient’s overall rating of change as the anchor. However, the response shift effect could differentially impact on the results of the MID depending on the anchor used. Future studies are warranted to investigate this possible risk of bias and take it into account in the MID determination [15].

Several terminologies were used to identify the MID. However, using a standardized term and acronym referring to the MID should be investigated in future studies to avoid variability in terminology and to obtain more accurately the maximum number of articles needed for future analysis.

A frequent limitation was the small sample size in the anchor categories. If the number of patients in each category of anchor is not sufficient, then the resulting MID cannot be reliable and the robustness in this case is thus questionable. Only one study determined the sample size specifically for the MID analysis. This sample size calculation must be systematically performed for MID determination, even if the analysis is not the primary objective of the study, for example when data from a randomized clinical trial are used. Calculating an appropriate sample size per category will ensure the robustness of the results. Furthermore, the occurrence of missing data can also bias the MID analysis. Therefore, it is also important to determine the profile of missing data, and consider imputing missing data using the appropriate method.

Using only one electronic database (PubMed searches) was the main limitation of this work. Unfortunately, due to lack of resource, we could not use other databases to perform this review. A risk of bias could thus be observed since other interesting papers may not be captured in this database. Hopefully, a manual research conducted to the same papers obtained via our algorithm in Pubmed.

This review must be expanded in future studies to address all methods that have been used to determine the MID either if they are including or not including in distribution-based or anchor-based methods (i.e. minimal detectable change, Receiver operating characteristic (ROC) curve, Item Response Theory, etc.).

In light of these results, greater attention should be paid to the methodology in future studies investigating the MID of a given PRO questionnaire, in order to ensure reliable results. This will also make it possible to use the MID for sample size determination when designing clinical trials with HRQOL or PRO as a primary endpoint, as well as for facilitating interpretation of the results. In the context of clinical trials in oncology, the MID is rarely used to interpret results in a clinically meaningful way. In a recent review of phase III trials in non-small cell lung cancer including a PRO endpoint, only 20% of studies interpreted the results in light of the MID [65]. The time-to-HRQOL-deterioration is a recently proposed method to analyze longitudinal HRQOL data [66]. One advantage of this method is to incorporate the MID in the definition of the event to qualify the deterioration. This guarantees the clinical significance of the results, but the choice of the MID is crucial since it has a direct impact on the results of the analysis.

## Conclusions

Further research is mandatory to improve the quality of the methodology used to determine the MID in HRQOL questionnaires used in oncology. In particular, the choice of an appropriate anchor(s) when using the anchor-based approach, or appropriate criteria when using the distribution-based approach is essential. The sample size should also be taken into account to produce reliable results. This could increase the use of these specific thresholds in future studies.

## Additional file

Additional file 1: **Table S1.** Information about Minimal important difference determination of the most used questionnaires (EORTC QLQ-C30 and FACT) (DOCX 32 kb)

## Abbreviations

EORTC: European organization for research and treatment of cancer
FACT: Functional assessment of cancer therapy
HRQOL: Health-related quality of life
MID: Minimally important difference
MMSE: Mini-Mental State Examination
PRO: Patient-reported outcome
SD: Standard deviation
SEM: Standard error of measurement
