# Supplementary material for: Distribution- and anchor-based methods to determine the minimally important difference on patient-reported outcome questionnaires in oncology: a structured review
Source: Health Qual Life Outcomes. 2018 Dec 11;16:228. doi: 10.1186/s12955-018-1055-z (PMC6288886; doi:10.1186/s12955-018-1055-z)
Supplement: Supplementary file 1 — Table S1. Information about Minimal important difference determination of the most used questionnaires (EORTC QLQ-C30 and FACT) (DOCX 32 kb) [file 12955_2018_1055_MOESM1_ESM.docx]

Supplementary Table 1. Information about Minimal important difference determination of the most used questionnaires (EORTC QLQ-C30 and FACT)

| PRO questionnaires | Cancer sites | Reference | Applied methods | Criteria reported for distribution-based method | Distribution-based MID (min – max) | Anchor if anchor-based method | Anchor-based MID for deterioration (min – max) | Anchor-based MID for improvement (min – max) | Number of patients  (N) | Minimum N by anchor category |
| --- | --- | --- | --- | --- | --- | --- | --- | --- | --- | --- |
| EORTC QLQ-C30 | All cancer sites | Kemmler, G. (2010) | Distribution and anchor-based | 1 SD, SEM | 5.3 – 27.7 | PRG | Unclear | Unclear | 160 | NR |
|  |  | Hong, F. (2013) | Anchor-based | NA | NA | PRG | 5.7 – 11.8 | 2.7 – 6.9 | 627 | NR |
|  | All advanced cancer | Bédard, G. (2014) | Distribution and anchor-based | 0.2 SD 0.3 SD, 0.5 SD and SEM | 1 – 16.8 | Global HRQOL dimension of the QLQ-C30 | 0.3 - 13.5 | 1.5 – 23 | 367 – 369 | 17 |
|  | Breast | Lemieux, J. (2007) | Distribution-based | 0.2 SD, 0.5 SD and SEM | 4.1 – 12.4 | NA | NA | NA | 133 | NA |
|  |  | Ousmen, A. (2016) | Anchor-based | NA | NA | PRG | 0.5 – 26 | 0 – 15.1 | 74 – 260 | 11 |
|  | Bone metastases | Zeng, L. (2012) | Distribution and anchor-based | 0.2 SD, 0.3, 0.5 SD and SEM | 2.1 – 18.7 | Performance status | 1 – 22.4 | 2.2 – 30.5 | 88 – 93 | 7 |
|  |  | Raman, S. (2018) | Distribution and anchor-based | 0.2 SD, 0.3 SD and 0.5 SD | 3.7 – 16.7 | Global HRQOL dimension of the QLQ-C30 | 9 – 24.2 | 0 – 13.3 | 360 – 375 | 90 |
|  | Oral cavity and oropharynx | Binenbaum, T. (2014) | Distribution-based | 0.5 SD | 6.5 – 14.4 | NA | NA | NA | 329 – 631 | NA |
|  | Brain | Maringwa, J.T (2011) | Distribution and anchor-based | 0.2 SD, 0.3, 0.5 SD and SEM | 4.4 – 16.7 | Performance status, MMSE | 1.8 – 12.3 | 5.6 – 14.3 | 420 – 572 | 26 |
|  | Lung | Maringwa, J.T (2011) | Distribution and anchor-based | 0.2 SD, 0.5 SD and SEM | 4 – 17 | Performance status, weight change | 3 – 11 | 2 – 16 | 410 – 519 | 38 |
|  |  |  |  |  |  |  |  |  |  |  |
|  |  |  |  |  |  |  |  |  |  |  |
| FACT-M | Melanoma | Askew, R.L. (2009) | Distribution and anchor-based | 0.2 SD, 0.3 SD and SEM | 2 – 3.8 | Performance status | 1.9 – 4.6 | 2.6 – 3.4 | 163 | 6 |
| FACT-M | Merkel cell carcinoma | Bharmal, M. (2017) | Distribution and anchor-based | 0.2 SD, 0.5 SD and SEM | 1.8 – 4.5 | Percentage change in tumor size | 2.9 | 2.7 | 70 | 7 |
| FACT-P | Prostate | Cella, D. (2009) | Distribution and anchor-based | 0.3 SD, 0.5 SD and SEM | 2.1 – 4.1 | Performance status | 2 – 3^a^ | | 809 | NR |
| FACT-AN | All cancer sites | Cella, D. (2002) | Distribution and anchor-based | 0.5 SD and SEM | 5.8 – 16.6 | Performance status; hemoglobin level | 6.5 – 28.3^a^ | | 50 – 2402 | 9 |
| FACT-L | Lung | Cella, D. (2002) | Distribution and anchor-based | 0.3 SD, 0.5 SD and SEM | 1.5 – 2.9 | Best overall response to treatment, time to disease progression | 1.2 – 1.9 | 0 – 2.4 | 573 | NR |
| FACT-Cog | Breast | Cheung, Y.T. (2014) | Distribution and anchor-based | 0.3 SD, 0.5 SD and SEM | 6.9 – 10.6 | Cognitive dimension of the QLQ-C30 | 9.6 | 2.2 | 220 | 8 |
| FACT-Lung Symptom Index-12 | Lung | Eton, D.T. (2007) | Distribution and anchor-based | 0.3 SD, 0.5 SD and SEM | 2.1 – 3.9 | Performance status | 3 – 5.6 | 3.3 – 8.1 | 91 | 8 |
| FACT-BRM | Kidney | Eton, D.T. (2006) | Distribution and anchor-based | 0.3 SD, 0.5 SD and SEM | 2.1 – 3.6 | Clinical distinct groups using performance status | 3.9 – 4.6 | 1.9 – 3.6 | 209 | 30 |
| FACT-B | Breast | Eton, D.T. (2004) | Distribution and anchor-based | 0.3 SD, 0.5 SD and SEM | 6.2 – 9.3 | Performance status, physician assessment of current pain, and response to treatment | 7 – 8^a^ | | ‘  128 – 643 | NR |
| FACT-F | All cancer sites | Mouysset, J.L. (2016) | Anchor-based | NA | NA | VAS of fatigue | NR | 3.5 | 510 | NR |
| FACT-HEP | Hepatobiliary | Steel, J. L. (2006) | Distribution and anchor-based | 0.3 SD, 0.5 SD and SEM | 7.7 – 17.6 | Alpha-fetoprotein, alkaline phosphate and hemoglobin levels, survival | 8 – 9^a^ | | 158 | NR |
| FACT-BRM | Myelogenous leukemia | Yost, K.J. (2005) | Distribution and anchor-based | SEM | 4.2 | Patient's rating of change; performance status | 5.5 – 28.2 | 3.6 – 9.9 | 144 – 164 | 11 |
| FACT-C | Colon | Yost, K.J. (2005) | Distribution and anchor-based | 0.3 SD, 0.5 SD and SEM | 4.9 – 8.5 | Performance status | 4.5 | 1.3 | 60 – 568 | 32 |

SD: Standard deviation, NA: Not applicable, PRG: Patient's rating of change, a: No distinction between deterioration and improvement, NR: Not reported

.
